# Case Report: Embolus in transit vs. *in situ* PFO thrombus

**DOI:** 10.3389/fcvm.2025.1721995

**Published:** 2026-01-12

**Authors:** Bijing Li, Haoyuan Wang, Huan Cen, Sinan Chen, Shengchun Shu, Bo Peng, Pengtao Sun

**Affiliations:** 1 Department of Ultrasonography, The Second Affiliated Hospital of Guangzhou University of Chinese Medicine, Guangzhou, China; 2 Department of Anesthesiology, Guangdong Provincial People’s Hospital (Guangdong Academy of Medical Sciences), Southern Medical University, Guangzhou, China; 3 Department of Cardiovascular Surgery, The Second Affiliated Hospital of Guangzhou University of Chinese Medicine, Guangzhou, China

**Keywords:** antithrombin deficiency, echocardiography, embolus in transit, *in situ* thrombus, paradoxical embolism, patent foramen ovale, thrombophilia

## Abstract

A crossing patent foramen ovale (PFO) thrombus is a thrombus that straddles both atria through a PFO, also called a transseptal thrombus or an impending paradoxical embolism. Although rare, this condition represents a highly critical clinical emergency. Clinically, such thrombi are usually classified as primary intracardiac (*in situ*) thrombosis or emboli in transit from the venous system. We report two contrasting cases of a thrombus straddling the foramen ovale documented with high-quality multimodality imaging and serial transesophageal echocardiography (TEE) during follow-up. Case 1 involved a 21-year-old man who presented with sudden severe dyspnea, profuse sweating, and transient loss of consciousness after a long-distance train journey. TEE demonstrated a large, highly mobile thrombus straddling the PFO with right heart enlargement and pulmonary hypertension, and CT pulmonary angiography (CTPA) confirmed extensive pulmonary embolism. During emergency surgery, thrombi were removed from the right atrium, PFO, left atrium, and pulmonary arteries with concomitant PFO closure. Subsequent targeted genetic testing revealed a heterozygous SERPINC1 nonsense variant classified as likely pathogenic for antithrombin deficiency, suggesting underlying hereditary thrombophilia. Case 2 involved a 75-year-old woman with hypertension and persistent atrial fibrillation who underwent TEE screening before planned catheter ablation, which revealed a small, relatively fixed thrombus confined to the PFO tunnel. She was managed conservatively with 20 mg of rivaroxaban once daily, and serial TEE at 54 and 141 days revealed progressive thrombus regression without peripheral embolic events. These cases illustrate typical imaging features and clinical contexts that help distinguish an embolus in transit from a presumed *in situ* PFO thrombus and show how careful determination of the thrombus origin and nature can guide individualized management, help prevent catastrophic embolic events, and improve patient outcomes.

## 1 Introduction

A thrombus at the foramen ovale is clinically important because it is associated with a high risk of a venous thrombus crossing a patent foramen ovale (PFO) into the arterial circulation (paradoxical embolism), potentially leading to ischemic stroke, transient ischemic attack (TIA), or peripheral arterial embolism. Recent studies (1, 2) have demonstrated that a PFO is not merely a passive “conduit for emboli” but may also serve as a site for thrombus formation. Once a thrombus becomes lodged at the foramen ovale (also referred to as a straddling thrombus or impending paradoxical embolism), it represents a life-threatening emergency that often requires urgent surgical or interventional management. The way in which a PFO thrombus is classified—either as an embolus in transit from the venous system or as an *in situ* intracardiac thrombus—directly influences the treatment strategy. Several case reports and small series have described thrombi straddling a PFO arising from these two mechanisms, but important uncertainties remain regarding their imaging features and clinical implications. Given this background, we describe two illustrative cases—one representing an embolus in transit and the other most compatible with *in situ* thrombus formation—and use multimodality imaging and longitudinal follow-up to highlight practical diagnostic clues and management considerations.

## 2 Case report

### 2.1 Case 1

**Figure 1:**
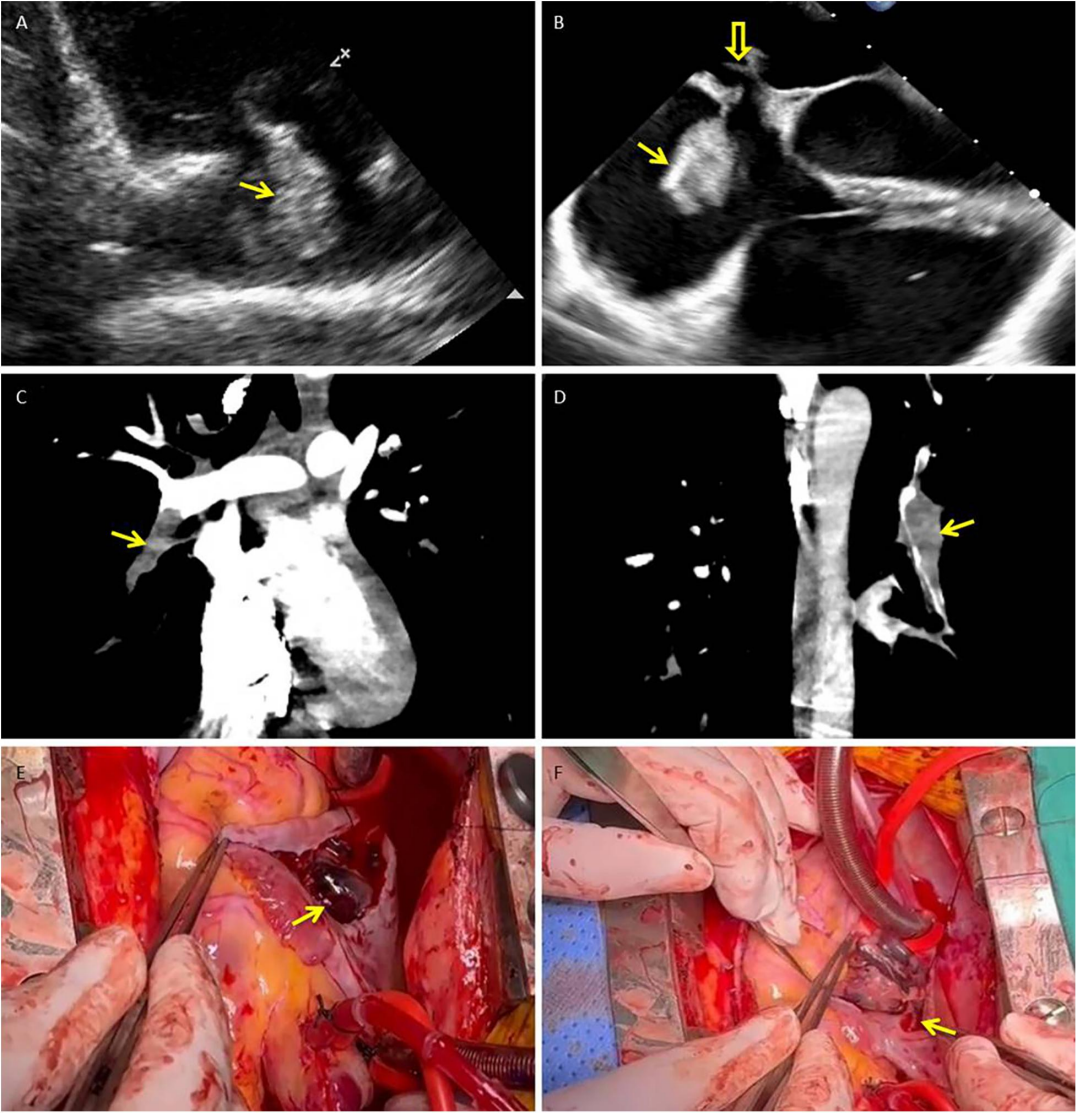
Thrombus in transit straddling a patent foramen ovale with concurrent pulmonary embolism: multimodality documentation and operative retrieval. **(A)** Transthoracic echocardiography (right ventricular inflow view) shows a highly mobile, serpiginous thrombus within the right atrium (arrow). **(B)** Transesophageal echocardiography (mid-esophageal four-chamber view) demonstrates the thrombus straddling the PFO (solid arrow), with its distal segment prolapsing into the left atrium (hollow arrow); right-sided chamber enlargement is present. **(C,D)** CT pulmonary angiography (coronal reformations) reveals acute pulmonary emboli, including an intraluminal filling defect in the right lower-lobe pulmonary artery **(C)** and additional defects in distal branches of the left pulmonary artery **(D)** (arrows). **(E,F)** Intraoperative photographs: **(E)** shows the large, friable thrombus (measuring approximately 2.0 × 8.5 cm) immediately after the right atrium was opened (arrow). **(F)** demonstrates the narrow stalk (approximately 3 mm) of the thrombus arising from the PFO ostium (arrow). PFO, patent foramen ovale; RA, right atrium; LA, left atrium; RV, right ventricle; TTE, transthoracic echocardiography; TEE, transesophageal echocardiography; CTPA, CT pulmonary angiography.

A 21-year-old man with sudden-onset severe dyspnea, profuse sweating, and transient loss of consciousness presented at our emergency department in May 2025. The day before symptom onset, he had undergone a long-distance train journey with prolonged immobilization. His medical history included a right lower leg fracture treated with internal fixation in 2020 and cerebral infarction secondary to cerebral venous thrombosis in 2023. After recovery from cerebral venous thrombosis, he was prescribed oral rivaroxaban (15 mg once daily) for secondary prevention but self-discontinued this medication approximately one year before admission, without medical advice. On admission, laboratory tests revealed markedly elevated levels of D-dimer (50.43 mg/L FEU; reference 0.00–0.50) and fibrinogen degradation products (>124.00 mg/L; reference 0.00–5.00). His oxygen saturation was 85% on room air. Transthoracic echocardiography (TTE) revealed a highly mobile, serpiginous mass within the right atrium (RA) consistent with a thrombus in transit, measuring approximately 35 × 22 mm (Figure 1A). Transesophageal echocardiography (TEE) revealed a moderately echogenic thrombus straddling the patent foramen ovale (PFO) and prolapsing into the left atrium (LA), accompanied by right-heart enlargement and pulmonary hypertension (Figure 1B). CT pulmonary angiography (CTPA) confirmed extensive bilateral pulmonary embolism (Figures 1C,D). Cerebral angiography revealed a patent intracranial arterial tree without hemorrhage or new embolic occlusion. Preoperative bilateral lower extremity venous Doppler ultrasonography did not reveal residual deep vein thrombosis.

The patient underwent emergency surgery. Through a right atriotomy, a friable thrombus measuring approximately 2.0 × 8.5 cm was removed from the RA; a narrow stalk (∼3 mm) appeared to originate from the PFO ostium (Figures 1E,F). Additional dark thrombi were evacuated from the LA and the left upper, lower, right middle, and lower pulmonary arteries. Surgical closure of the PFO was performed, and a temporary inferior vena cava (IVC) filter was implanted at the end of the procedure as a prophylactic measure to reduce the risk of early recurrent embolism during the perioperative period, when full-dose anticoagulation needed to be carefully re-introduced. Postoperatively, low molecular weight heparin was started on postoperative day 1, followed by a transition to warfarin with an overlap period. Warfarin was continued until hospital discharge, after which long-term secondary prevention was maintained with rivaroxaban once daily. Histopathological examination confirmed organized thrombi in the RA, LA, and pulmonary arterial specimens. Follow-up TTE on postoperative days 15 and 29 revealed no residual intracardiac thrombus and no echocardiographic evidence of right heart strain. Subsequently, a targeted next-generation sequencing panel for hereditary cerebrovascular disease identified a heterozygous nonsense variant in SERPINC1 (c.51T > A, p.Tyr17*), classified as likely pathogenic for antithrombin III deficiency, together with missense variants in PROC (c.629C > T, p.Pro210Leu) and MTHFR (c.665C > T, p.Ala222Val; c.136C > T, p.Arg46Trp; c.700G > A, p.Asp234Asn). According to the laboratory report, only the SERPINC1 variant was considered likely pathogenic, whereas the other variants were of uncertain or modest clinical significance.

### 2.2 Case 2

**Figure 2:**
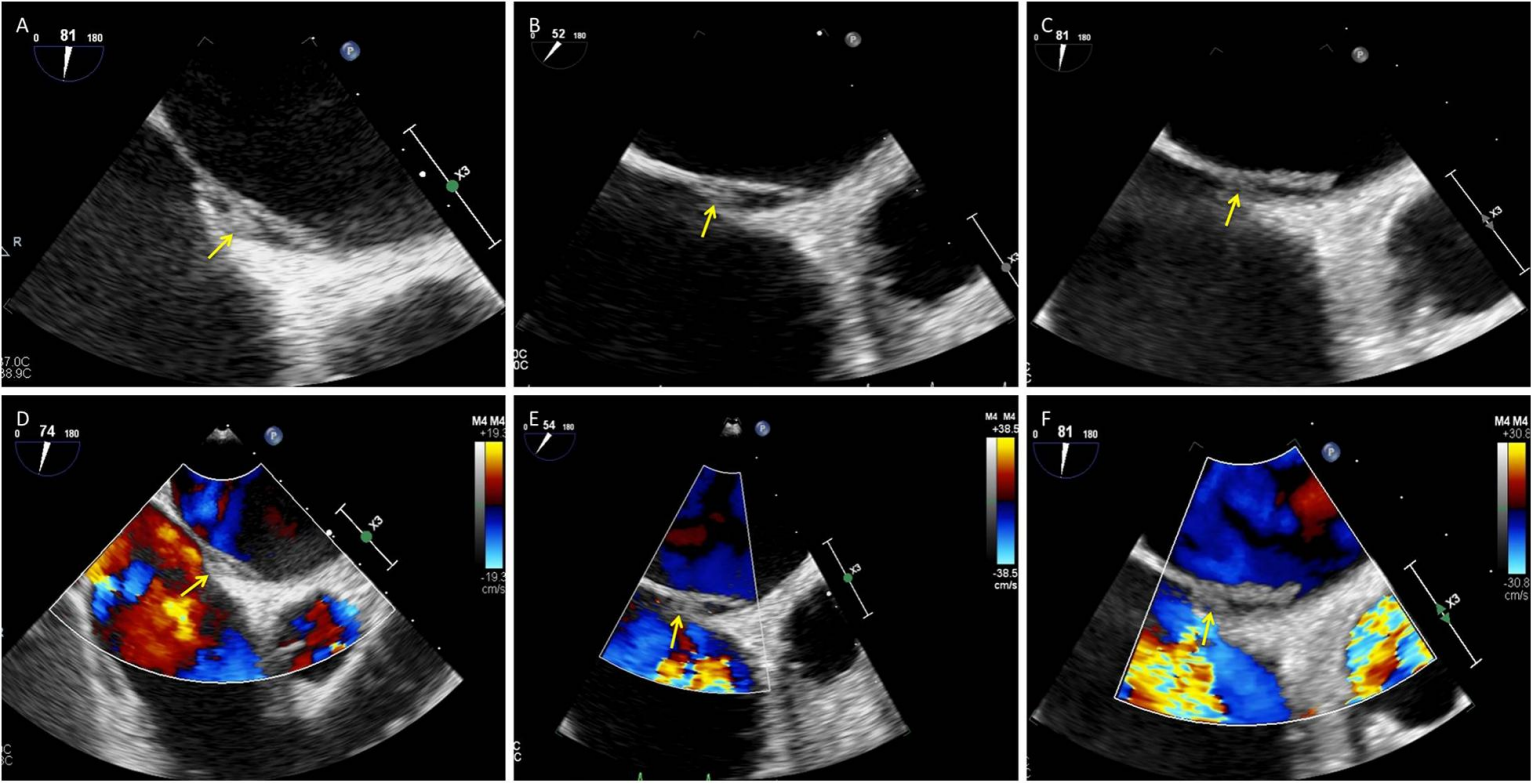
Small presumed *in situ* thrombus within the PFO tunnel with interval regression under oral anticoagulation: serial TEE. **(A–C)** Grayscale TEE images (mid-esophageal views) obtained at baseline, day 54, and day 141 show a small, relatively fixed thrombus within the PFO tunnel (arrows). The thrombus measured 3.1 × 9.1 mm at baseline **(A)**, 2.3 × 5.6 mm on day 54 **(B)**, and 2.1 × 4.6 mm on day 141 **(C)**. **(D–F)** Corresponding color Doppler images at the same time points depict interatrial flow across the PFO (arrows). PFO, patent foramen ovale; TEE, transesophageal echocardiography; RA, right atrium; LA, left atrium.

A 75-year-old woman was admitted with a chief complaint of recurrent chest tightness for more than 3 years, which had worsened over the two days prior to admission, accompanied by shortness of breath and dizziness. Her medical history included hypertension for more than 20 years and diabetes for more than 2 years. Laboratory findings included an N-terminal pro–B-type natriuretic peptide (NT-proBNP) concentration of 1,605 ng/L (0–125), a hypersensitive troponin concentration of 0.014 µg/L, a D-dimer concentration of 0.51 mg/L FEU (0–0.5), and an electrocardiogram demonstrating persistent atrial fibrillation (AF) with ST–T abnormalities. Cranial magnetic resonance imaging (MRI) revealed small chronic ischemic infarcts in the right corona radiata and bilateral centrum semiovale, with no evidence of acute infarction. TTE revealed biatrial enlargement, mild mitral regurgitation, moderate tricuspid regurgitation, borderline pulmonary hypertension, and normal left ventricular systolic function. Transesophageal echocardiography (TEE) was performed to exclude intracardiac thrombus prior to planned radiofrequency catheter ablation; importantly, no thrombus was detected in either the left atrial appendage or the right atrial appendage. Initially, TEE revealed separation of the primary and secondary septum, with a fixed, slender, medium-echo thrombus measuring approximately 3.1 × 9.1 mm straddling the PFO (Figures 2A,D). Spontaneous echocardiographic contrast in both atria suggested blood stasis. Anticoagulation therapy with 20 mg rivaroxaban once daily was immediately initiated. TEE performed 54 days later revealed a thrombus measuring approximately 2.3 × 5.6 mm (Figures 2B,E). Final TEE performed 141 days later revealed a thrombus measuring 2.1 × 4.6 mm (Figures 2C,F); moreover, no peripheral embolic events occurred during the treatment period. The thrombus decreased in size following oral anticoagulation treatment with rivaroxaban.

## 3 Discussion

A PFO is present in approximately 25% of adults (3). In most individuals, it remains asymptomatic, but it can provide a channel for venous thrombi to enter the systemic circulation and cause paradoxical embolism with serious sequelae such as stroke or peripheral arterial embolism. From a mechanistic perspective, PFO-associated thrombi are usually considered to arise either as primary intracardiac (*in situ*) thrombi within or near the PFO tunnel or as emboli in transit that originate from the venous system (e.g., deep vein thrombosis, DVT) and become temporarily trapped at the PFO (4, 5). Distinguishing between these mechanisms is clinically important because it influences both acute management and long-term secondary prevention.

In Case 1, TTE and TEE demonstrated a large, highly mobile serpiginous thrombus within the RA that straddled the PFO and prolapsed into the LA, accompanied by right heart enlargement and pulmonary hypertension—typical features of an embolus in transit. Migratory PFO thrombi are usually elongated, with few attachment points and marked excursion; they can be seen drifting between the atria during the cardiac cycle (6–8). They often occur in younger or middle-aged patients who present abruptly with chest pain, dyspnea, hypoxemia, syncope, or shock in the context of acute venous thromboembolism (9, 10). Our patient had a history of right lower leg fracture and prior cerebral venous thrombosis with ischemic stroke, had recently undergone a long-distance train journey with prolonged immobilization, and presented with massive pulmonary embolism on CTPA. Although preoperative ultrasound did not reveal residual DVT, the combination of a large free-floating RA thrombus straddling the PFO, massive pulmonary embolism, and this thrombotic history strongly supported the diagnosis of an embolus in transit originating from the peripheral venous system. His subsequent genetic testing demonstrated a likely pathogenic SERPINC1 nonsense variant (antithrombin III deficiency), further suggesting that this extreme presentation occurred on a background of hereditary thrombophilia and venous thromboembolism susceptibility. In this context, emergency surgical removal of thrombi from the RA, PFO, and pulmonary arteries, combined with PFO closure and temporary IVC filter placement, was considered the most appropriate strategy to prevent further embolization.

In contrast, *in situ* thrombi are generally smaller, less mobile, and more closely adherent to the interatrial septum or PFO tunnel. They tend to occur in patients with underlying cardiac disease or atrial factors such as AF or structural abnormalities and may lack an obvious peripheral venous source (6, 11). In Case 2, a 75-year-old woman with hypertension and persistent AF had biatrial enlargement and spontaneous echocardiographic contrast, indicating pronounced atrial blood stasis. TEE revealed separation of the primary and secondary septa and a small, relatively fixed, slender thrombus within the PFO tunnel that gradually regressed under oral anticoagulation, without clinical evidence of pulmonary embolism or peripheral embolic events during 141 days of follow-up. These observations are more compatible with *in situ* thrombus formation within the PFO tunnel than with a large embolus in transit.

Nevertheless, the diagnostic work-up in Case 2 was incomplete: lower-extremity venous Doppler ultrasound, contrast-enhanced TTE/TEE with bubble study, and formal thrombophilia testing were not performed, and AF itself is a major confounder of cardioembolism. A venous source and paradoxical embolism, therefore, cannot be definitively excluded, and our classification of this lesion as most compatible with *in situ* PFO thrombus should be regarded as presumptive rather than conclusive. From a practical standpoint, signs of acute pulmonary embolism, lower extremity DVT, right ventricular pressure overload, and a large, highly mobile serpiginous thrombus spanning both atria favor an embolus in transit. Conversely, small, relatively fixed thrombi confined to the PFO tunnel in patients with AF or other prothrombotic cardiac conditions, in the absence of clear peripheral venous triggers, are more suggestive of *in situ* formation—but as illustrated by Case 2, such distinctions are sometimes probabilistic rather than definitive.

Echocardiography plays a central role in this setting. Contrast-enhanced TTE or TEE with intravenous microbubble injection (“bubble study”) can document right-to-left shunting across a PFO and grade the shunt. TEE provides higher spatial resolution than TTE does, allows direct visualization of the PFO tunnel and associated structures (such as atrial septal aneurysm or a Chiari network), and is therefore crucial for characterizing thrombus morphology and attachment and for planning potential interventional or surgical procedures (12). TTE may detect large thrombi that prolapse into the LA, but it has limited sensitivity for detecting small or tunnel-confined thrombi. In a systematic review, approximately 12.7% of PFO straddling thrombi were missed by TTE (13, 14), underscoring its lower sensitivity than that of TEE. Thus, when clinical features suggest concomitant pulmonary and systemic embolism, TEE should be performed promptly even if the TTE result is unremarkable.

Once a foramen ovale thrombus is diagnosed, rapid therapeutic decision-making is needed because of the high risk of catastrophic embolization (15, 16). Available treatment strategies include conservative anticoagulation, systemic thrombolysis, and surgical or percutaneous thrombectomy with PFO closure. Each approach has advantages and limitations, and management should be individualized on the basis of hemodynamic stability, thrombus size and morphology, comorbid conditions, bleeding risk, and local expertise (17, 18). Our two cases illustrate how multimodality imaging can guide divergent management decisions: urgent surgical embolectomy and PFO closure with temporary IVC filter protection in a young patient with a large embolus in transit and hereditary thrombophilia vs. anticoagulation alone with structured imaging follow-up in an elderly patient with a small, presumed *in situ* PFO thrombus and significant diagnostic uncertainty. Although PFO thrombosis is rare, clinicians should maintain a high index of suspicion in patients with AF or a history of venous thromboembolism, and early echocardiographic assessment is essential to optimize outcomes.

This study has several limitations. First, comprehensive hypercoagulability (thrombophilia) testing was not completed for either patient. In Case 1, a postevent targeted next-generation sequencing panel for hereditary cerebrovascular disease genes revealed a heterozygous SERPINC1 nonsense variant (c.51T > A, p.Tyr17*), classified as likely pathogenic for antithrombin III deficiency, together with additional variants of uncertain or modest clinical significance in PROC and MTHFR. However, functional assays and a full laboratory thrombophilia work-up (including antithrombin activity, protein C and S levels, antiphospholipid antibodies, and factor V Leiden/prothrombin gene testing) were not performed, which limits our ability to characterize the underlying thrombotic diathesis fully and to draw definitive conclusions regarding the etiology of the thrombi. Second, in Case 2, lower extremity venous Doppler ultrasound, contrast-enhanced TTE/TEE bubble studies and transcranial Doppler bubble monitoring were not performed, and the coexistence of atrial fibrillation may further confound the attribution of the thrombus to the PFO vs. another cardiac or venous source. Consequently, a venous source and paradoxical embolism cannot be definitively excluded in Case 2, and our classification of this lesion as most compatible with *in situ* PFO thrombus should be interpreted with caution and regarded as hypothesis-generating rather than conclusive. Future management of similar cases should therefore incorporate more comprehensive diagnostic work-ups and systematic follow-up.

## 4 Conclusions

Foramen ovale thrombus is a rare but potentially life-threatening clinical entity with diverse formation mechanisms; by reporting two illustrative cases—a large embolus in transit and a small, presumed *in situ* PFO thrombus—we highlight how clinical context together with detailed transthoracic and transesophageal echocardiographic assessment can help distinguish between these pathophysiological pathways and guide individualized management, ranging from emergency surgical embolectomy to anticoagulation alone with structured imaging follow-up. The identification of a heterozygous SERPINC1 nonsense variant consistent with congenital antithrombin deficiency in our young patient further supports the consideration of hereditary thrombophilia in similar presentations of paradoxical embolism or recurrent venous thromboembolism, although functional assays and a comprehensive laboratory thrombophilia work-up remain essential. Conversely, our elderly patient with persistent atrial fibrillation and an incomplete diagnostic work-up illustrates that mechanistic classification (*in situ* vs. embolus in transit) is sometimes probabilistic rather than definitive and must be interpreted with caution. Overall, careful integration of the thrombotic risk profile with high-quality multimodality imaging by a multidisciplinary team is fundamental for optimizing treatment plans, preventing catastrophic embolic events, and improving patient outcomes.

## Data Availability

The original contributions presented in the study are included in the article/Supplementary Material, further inquiries can be directed to the corresponding author.
